# Supplementary material for: Access of older people to primary health care in low and middle-income countries: A systematic scoping review
Source: PLoS One. 2024 Apr 19;19(4):e0298973. doi: 10.1371/journal.pone.0298973 (PMC11029620; doi:10.1371/journal.pone.0298973)
Supplement: S1 File — (DOCX) [file pone.0298973.s002.docx]

## Additional reported and summarized data

## Defining and measuring access and other related concepts

Healthcare access was defined only in six included studies [27,28,31,34,38,53]. Those definitions varied and focused either on access dimensions [31,38,53], the capacity of the health system to supply services [28], or the utilization of healthcare services during a timespan or based on a perceived need [27,34,53]. Four studies suggested four different indicators to measure healthcare access [28,38,41] as presented in table A. One of the alternative concepts to access that was commonly measured, is the utilization of PHC services. Utilization indicators measured the number of times, a specific PHC service was used during a specific timespan. Services used and time spans varied across studies; outpatient or homecare [44,45,47,53], medical visits [33,35,37, 41,42,48], and screening tests [37], are examples of services which use was measured within a timespan varying from 4 months to two years. In addition to healthcare utilization, two studies focused on equity of access [46] and accessibility [49] as main concepts. All definitions and indicators are compiled in table A also showing that, existing theoretical frameworks guided the data collection and analysis of only seven studies on healthcare access and utilization. Three of them used the conceptual model of Health Service Utilization of Aday and Andersen that is commonly adopted in similar studies [15]. Results are consistent with those of Levesque et al., [17] and Carroll et al. [15] who highlighted that healthcare access is conceptualized differently in the literature with healthcare utilization being the commonly measured concept especially in quantitative studies.

| Table A. Conceptualization of access and other relevant concepts | | | |  | |  |
| --- | --- | --- | --- | --- | --- | --- |
| Study ID/ Country | Study Design | Definition and measurement of Access | Definition and measurement of other relevant concepts | | Theoretical Framework | |
| Amorim 2020 Brazil | Cross sectional | None | None | | None | |
| Augusto 2019 Brazil | Cross sectional | None | *Indicator for PHC use:* at least one medical visit in the last 12 months | | Starfield's Theoretical Framework to assess  health services utilization | |
| Blay 2008 Brazil | Cross sectional | *Definition*: self-report of any outpatient visits in the previous 6 months, any hospitalization within the previous 12 months, and among the hospitalized whether there had been one or more than one hospitalization | None | | None | |
| Bos 2007 Brazil | Cross sectional | None | *Indicator for PHC use:* at least one medical visit in the past 6 months prior to the study | | None | |
| Caner 2019 Turkey | Cross sectional | None | None | | None | |
| Carreira 2010 Brazil | Qualitative | None | None | | None | |
| Cesário 2021 Brazil | Cross sectional | *Definition*: The user's entry into the health service according to the individual's established need. | None | | None | |
| Ferreira 2020 Brazil | Cross sectional | *Definition*: Access meaning is commonly associated with health system performance related to their supply capacity. | *Indicator for PHC use:*  the first service that the person seeks when in need for healthcare (reference service) | | Conceptual model of health service  utilization of Aday and Andersen | |
| Garcia-Ramirez 2020 Colombia | Cross sectional | None | *Indicator for PHC use*:  - Preventive care: use of pap smear/ mammogram/ prostate cancer screening in the last 2 years  - Outpatient care: medical visit in the last 4 months; visit to any other health professional in the last year | | None | |
| Girondi 2011 Brazil | Integrative review | None | None | | None | |
| Gu 2009 China | Longitudinal | *Definition*: consisting of availability, accessibility, accommodation, affordability, and acceptability as defined by Penchanski and Thomas (1981).  *Indicator:* whether a person can get adequate medical service when necessary. | None | | None | |
| Hu 2019 China | Cross sectional |  | *Indicator for accessibility:* distance and time to reach the nearest primary care facility | | Conceptual model of health service  utilization of Aday and Andersen | |
| Kelly 2019 South Africa | Qualitative | None | None included | | None | |
| Macinko 2018 Brazil | Longitudinal | *Indicator:* whether the person can get an appointment within 24 hours and whether this can be done through phone. | *Indicator for PHC use*: number of medical visits broken down into general practitioner (GP) and specialist visits in the past 12 months | | None | |
| Mahfouz 2004 Saudi Arabia | Cross sectional | None | *Indicator for PHC use*: average number of medical visits per person per year | | None | |
| Martins 2014 Brazil | Review of documents Cross sectional | None | None | | None | |
| Motsohi 2020 South Africa | Qualitative | None | None | | None | |
| Nam 2020 Korea | Cross sectional | None | *Indicator for PHC use*: use of any PHC services in the last year | | None | |
| Nwakasi 2019 Ghana | Cross sectional | None | *Indicator for PHC use*: number of times an individual used outpatient care or home medical care that did not involve overnight stay at a healthcare facility in the past 12 months. | | The behavioral model for vulnerable  populations (explains health behaviors like  services utilization) | |
| Park 2012 South Korea | Cross sectional | None | *Indicator for equitable access*:  "Access is equitable to the extent that predisposing, need-related demographic factors such as age and gender, as well as illness, account for health care utilization". | | Conceptual model of health service  utilization of Aday and Andersen | |
| Paskulin 2011 Brazil | Cross sectional | *Definition*: the availability of health services and their adequate coverage and is directly related to the health-disease process. It includes geographic, functional, sociocultural, and economic aspects. | None | | None | |
| Peltzer 2012 South Africa | Cross sectional | None | *Indicator for PHC use*: use of an outpatient health service in the past 12 months. | | None | |
| Placideli 2020 Brazil | Cross sectional | None | None | | None | |
| Rodrigues 2009 Brazil | Cross sectional | None | *Indicator for PHC use*:  1) number of medical visits in the past 6 months at the healthcare unit that serve the area where a person lives 2) number of participations in groups of chronic conditions in the past year. | | None | |
| Santos 2020 Brazil | Cross sectional | None | Definition of Accessibility: "facility of movement, including physical and structural autonomy and safety of such spaces so as to enable access and use of urban infrastructure by all citizens, including those who have locomotion difficulties". | | None | |
| Ssensamba 2019 Uganda | Cross sectional | None | None | | Adapted from the WHO's building blocks  framework (evaluate the six critical pillars  for health service delivery) | |
| Thumé 2011 Brazil | Cross sectional | *Indicator:* whether the respondent had received a care at home from a health professional during the past three months (measuring access to home-care) | None | | None | |
| Yam 2019 Hong Kong | Cross sectional | None | None | | None | |
| Sreerupa 2017  Mnaymar | Mixed method | *Definition*: the self-reported utilisation of outpatient and inpatient healthcare. It consists of 4 dimensions: availability, geographic accessibility, affordability, and acceptability | *Indicator for PHC use*: healthcare needed during the preceding 12 months | | Jacobs et al comprehensive analytical  framework of barriers to access | |

## Services provided within PHC

Within PHC, a range of services are reported without being detailed in included studies, making comparisons of PHC packages across LMICs difficult. Eight studies did not indicate any description of PHC services [25,29,32,35,38,39,45,47] three studies mentioned broadly that PHC entail health promotion, disease prevention and rehabilitation services [26,28,37] while three studies, from Brazil, referred to services delivered through the Family Health Strategy without any further description [27,33,34]. Medical consultation was the most reported service across studies from all countries [26,31,36,37,40-44,46,52,53] and valued as the main indicator for access to, and use of PHC services. Reported PHC services from countries were those that do not require inpatient stay at the facility except for Uganda [50], where public primary health care services are delivered through different care-level facilities. Among reported services, the following ones seem to be particularly essential for older people according to their needs: dementia support [40,44], home visits [26,30,40,52], vaccination [31,44], cancer screening [37], treatment of chronic diseases [40,44] educational activities [26,48], Nursing care [52,53], Oral care [30,52], Physiotherapy [44] and medication supply [26,31,40,44,52].

Reported services lacked focus on preventive and geriatric care. However, older people with complex healthcare needs, require a set of preventive and geriatric services that are best delivered at the community level, through PHC settings [9]. This includes health education, vaccination, screenings, and comprehensive geriatric assessment to early detect and manage conditions that can come along with advanced age [9].

## Interventions to maximize access to PHC

Two studies [51,52] were conducted with the aim of assessing the impact of specific interventions on the increase in access and utilization of PHC services. In Honk Kong [52] the Healthcare Voucher Scheme successfully increased the use of PHC services. However, older people used curative services for acute conditions rather than preventive ones. In Brazil [51] the adoption of the Family Health Strategy (FHS) by several municipalities increased the access to, and utilization of home care and public services compared to municipalities that adhered to Traditional PHC. Moreover, homecare was identified as an effective strategy that allows a better management of health conditions and maximizes access of older people to services [25,26].

## Consultation Exercise

A consultation with older people is reported in the protocol and was not included in the reporting of this systematic review. The rationale for this was the delay in writing the protocol to completing the review – and the Lebanon was impacted by economic downturn, Beirut blast and COVID 19. It was not possible to gain ethical approval and arrange to meet with older people during this challenging period. This component will be part of the interviews in phase 2 of the research and questions developed for interviews and focus groups will be informed by this systematic scoping review.
