# Supplementary material for: Access of older people to primary health care in low and middle-income countries: A systematic scoping review
Source: PLoS One. 2024 Apr 19;19(4):e0298973. doi: 10.1371/journal.pone.0298973 (PMC11029620; doi:10.1371/journal.pone.0298973)
Supplement: Attachment — Submitted filename: PLOS ONE Older person access to PHC.docx [file pone.0298973.s005.docx]

**PLOS ONE**

**Title :-** Access of older people to primary health care in low and middle-income countries: a systematic scoping review –

1. **The adequacy of the design and thoroughness in the execution of the research:-**

The topic addresses the provision of access of older persons to Primary Health Care using a systematic review design and thematic approach in the data analysis. The topic is of public health interest. The design is adequate and appropriate. The thoroughness in the execution of the project is commendable.

2. **The Authors knowledge and use of existing literature on the subject:-** The literature is deem to be adequate, but there is the need to have a definition of terms with respect to the determinants of the outcome of interest.
3. **The specific contribution of the Dissertation to knowledge.**

The study contribution of this research is in highlighting the special services provided for these age groups who might be marginalised to health care services by the facilities and its’ effect on the quality of services provided.

4. **The literary style and technical presentation of the Dissertation.**

The literary style is fluent and readable.

**Final Statement:- The research work is recommended for publication as it is.**
