# Supplementary material for: Access of older people to primary health care in low and middle-income countries: A systematic scoping review
Source: PLoS One. 2024 Apr 19;19(4):e0298973. doi: 10.1371/journal.pone.0298973 (PMC11029620; doi:10.1371/journal.pone.0298973)
Supplement: Attachment — Submitted filename: Response to Reviewers.docx [file pone.0298973.s006.docx]

University College Dublin

Belfield, Dublin 4, Ireland.

T: +353 1 716 7777

December 23, 2023

Dear reviewers,

The co-authors and me, would like to thank you for the review and constructive feedback on our submitted manuscript (PONE-D-23-19442) entitled “Access of older people to primary health care in low and middle-income countries: a systematic scoping review”.

As advised, we revised the manuscript and addressed the points raised during the review process as shown in the below table:

| Editor comments | Response to reviewers |
| --- | --- |
| Ensure that the manuscript meets PLOS ONE's style requirements | Done as per the Track Changes version |
| PubMed strategy: include number of articles at each stage | Done |
| Prisma flow diagram: add reasons for rejecting 930 articles in the first screening phase | The title and abstracts for 1004 records were screened and 930 were excluded as they did not meet the inclusion criteria (mentioned in the Prisma flow diagram). Specific reasons for excluding on title and abstract screening is not a requirement for Cochrane systematic reviews and is not a requirement in the Cochrane Handbook – current version 2023 MECIR Box 4.6.c Relevant expectations for conduct of intervention reviews.  Title and abstract screening was completed by two reviewers working independently and a third reviewer was responsible for managing discrepancies before moving to full text review. |
| Amendments to the protocol mentioned in the letter to editor should be reflected in the methods | In the letter we mentioned that the research question was narrowed down. The protocol included 5 sub-questions. We cited clearly that data presented in the manuscript report on two main questions related to experiences of older people with access, and access barriers and enablers. Summarized data answering the remaining questions mentioned in the protocol are available in the S2 file of the supporting information (manuscript lines 359-376).  The removal of LILACS database is reported in line 380.  A consultation with older people is reported in the protocol and was not included in the reporting of this systematic review. The rationale for this was the delay in writing the protocol to completing the review – and the Lebanon was impacted by economic downturn, Beirut blast and COVID 19. It was not possible to gain ethical approval and arrange to meet with older people during this challenging period. This component will be part of the interviews in phase 2 of the research and questions developed for interviews and focus groups will be informed by this systematic scoping review. |
| Definition of terms with respect to the determinants of the outcome | Health care access is defined (line 137); primary health care is defined (line 74); older people defined (line 134). |
| The definition of "older people" adopted for this study should be stated in this and all other resultant publications from this study. The UN definition of older people is people over 60 years, but this study protocol demanded the recruitment of persons 60 years and above. | Older people are defined in line 134 of the manuscript. According to UN published reports mentioned below, older people are those aged 60 or over. We adopted this definition in the protocol and selected relevant studies accordingly.   1. United Nations Department of Economic and Social Affairs (UNDESA). World population aging; 2015. Page 1. Available from: <https://www.un.org/en/development/desa/population/publications/pdf/ageing/WPA2015_Report.pdf> 2. United Nations, Department of Economic and Social Affairs (UNDESA). Income Poverty in Old Age: an Emerging Development Priority [Internet]. Place unknown: UNDESA; 2019. Page 2. Available from: <https://www.un.org/esa/socdev/ageing/documents/PovertyIssuePaperAgeing.pdf> 3. United Nations Development Programme. Ageing, Older Persons, and the 2030 Agenda for sustainable development. (No date). Page 8. Available from: https://www.un.org/development/desa/dspd/wp-content/uploads/sites/22/2017/08/Ageing-Older-Persons-and-2030-Agenda_Issues-Brief-low-resolution-.pdf |
| Review reference list | Done |
| Upload figure files to PACE digital diagnostic tool to ensure they meet PLOS requirements | Done |

Kindly note that the reference list was checked and a few links to cited articles were updated.

Thank you for considering this updated version.

Sincerely,

Saydeh Dableh,

Doctoral student, stage 2

University College Dublin, School of Nursing, Midwifery and Health Systems

[Saydeh.Dableh@ucdconnect.ie](mailto:Saydeh.Dableh@ucdconnect.ie).

9613721639

Co-authors

Dr. Kate Frazer

Associate Professor

University College Dublin - School of Nursing, Midwifery and Health Systems

Kate.frazer@ucd.ie

Mr. Diarmuid Stokes

Librarian

University College Dublin, UCD Library

Diarmuid.stokes@ucd.ie

Pr. Thilo Kroll

Professor of Health Systems Management

University College Dublin - School of Nursing, Midwifery and Health Systems

[Thiol.kroll@ucd.ie](mailto:Thiol.kroll@ucd.ie)
